# 1,2-Diphenyl-1*H*-imidazo[4,5-*f*][1,10]phenanthroline

**DOI:** 10.1107/S1600536811010890

**Published:** 2011-03-26

**Authors:** S. Rosepriya, A. Thiruvalluvar, J. Jayabharathi, M. Venkatesh Perumal, R. J. Butcher, J. P. Jasinski, J. A. Golen

**Affiliations:** a PG Research Department of Physics, Rajah Serfoji Government College (Autonomous), Thanjavur 613 005, Tamilnadu, India; b Department of Chemistry, Annamalai University, Annamalai Nagar 608 002, Tamilnadu, India; c Department of Chemistry, Howard University, 525 College Street NW, Washington, DC 20059, USA; d Department of Chemistry, Keene State College, 229 Main Street, Keene, NH 03435-2001, USA

## Abstract

In the title compound, C_25_H_16_N_4_, the fused ring system is essentially planar [maximum deviation = 0.1012 (15) Å]. The imidazole ring makes dihedral angles of 77.41 (8) and 56.26 (8)° with the phenyl rings attached to nitro­gen and carbon, respectively. The dihedral angle between the two phenyl rings is 65.50 (8)°. Weak C—H⋯π inter­actions are found in the crystal structure.

## Related literature

For 1,2-diphenyl-1*H*-imidazo[4,5-*f*][1,10]phenanthroline derivatives, see: Hadadzadeh *et al.* (2006 ▶). For metal complexes of the 1,10-phenanthroline-5,6-dione ligand, see: Goss & Abruna (1985 ▶).
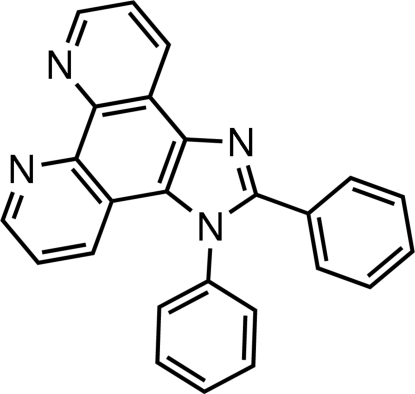


## Experimental

### 

#### Crystal data

C_25_H_16_N_4_
*M* *_r_* = 372.42
Triclinic, 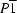

*a* = 8.8693 (7) Å
*b* = 10.0637 (6) Å
*c* = 11.8960 (9) Å
α = 100.219 (6)°
β = 110.310 (7)°
γ = 102.475 (6)°
*V* = 934.63 (14) Å^3^
*Z* = 2
Cu *K*α radiation
μ = 0.63 mm^−1^
*T* = 170 K
0.43 × 0.38 × 0.26 mm

#### Data collection

Oxford Diffraction Xcalibur Eos Gemini diffractometer
Absorption correction: multi-scan (*CrysAlis RED*; Oxford Diffraction, 2010 ▶) *T*_min_ = 0.965, *T*_max_ = 1.000
5832 measured reflections
3522 independent reflections
3121 reflections with *I* > 2σ(*I*)
*R*_int_ = 0.014

#### Refinement

*R*[*F* ^2^ > 2σ(*F* ^2^)] = 0.042
*wR*(*F* ^2^) = 0.117
*S* = 1.05
3522 reflections
262 parameters
H-atom parameters constrained
Δρ_max_ = 0.18 e Å^−3^
Δρ_min_ = −0.17 e Å^−3^

### 

Data collection: *CrysAlis PRO* (Oxford Diffraction, 2010 ▶); cell refinement: *CrysAlis PRO*; data reduction: *CrysAlis RED* (Oxford Diffraction, 2010 ▶); program(s) used to solve structure: *SHELXS97* (Sheldrick, 2008 ▶); program(s) used to refine structure: *SHELXL97* (Sheldrick, 2008 ▶); molecular graphics: *ORTEP-3* (Farrugia, 1997 ▶); software used to prepare material for publication: *PLATON* (Spek, 2009 ▶).

## Supplementary Material

Crystal structure: contains datablocks global, I. DOI: 10.1107/S1600536811010890/hg5009sup1.cif

Structure factors: contains datablocks I. DOI: 10.1107/S1600536811010890/hg5009Isup2.hkl

Additional supplementary materials:  crystallographic information; 3D view; checkCIF report

## Figures and Tables

**Table 1:** Hydrogen-bond geometry (Å, °) *Cg*4 and *Cg*6 are the centroids of the C4–C6/C11/C12/C17 and C24–C29 rings, respectively.

*D*—H⋯*A*	*D*—H	H⋯*A*	*D*⋯*A*	*D*—H⋯*A*
C15—H15⋯*Cg*6^i^	0.95	2.86	3.757 (3)	157
C25—H25⋯*Cg*4^ii^	0.95	2.75	3.4835 (16)	135

Symmetry codes: (i) 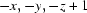; (ii) 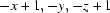.

**Fig. 1.**
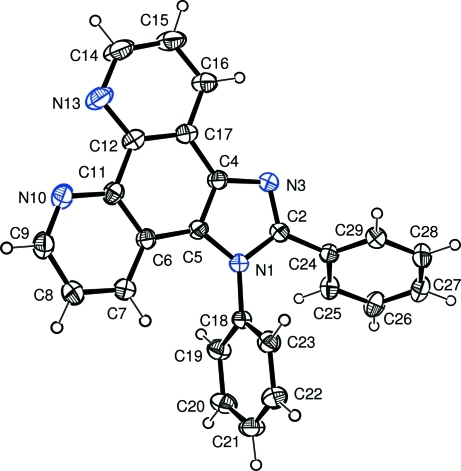
The molecular structure of the title compound, showing the atom-numbering scheme and displacement ellipsoids drawn at the 30% probability level. H atoms are shown as small spheres of arbitrary radius.

## supplementary crystallographic information

### Comment

1,2-diphenyl-1*H*-imidazo[4,5-*f*][1,10]phenanthroline derivatives play important roles as molecular scaffolding for supramolecular assemblies, building block for the synthesis of metallo-dendrimers, thin films of luminescent complexes and ligands for synthesis of ring opening metathesis polymerization (ROMP) (Hadadzadeh *et al.*, 2006). Metal complexes of phen-dione ligand allow for the variation and control of redox properties over a wide range as well as the fine tuning of potentials through pH changes (Goss & Abruna, 1985). Since our group doing the research in organic light emitting devices, we are interested to use the title compound as ligand for synthesizing Ir(III) complexes.

In the title compound (Fig. 1), C_25_H_16_N_4_, the fused ring system is essentially planar [maximum deviation of 0.1012 (15) Å for N13]. The imidazole ring makes dihedral angles of 77.41 (8) and 56.26 (8)° with the phenyl ring (C18—C23) attached to N1 and phenyl ring (C24—C29) attached to C2 respectively. The dihedral angle between the two phenyl rings is 65.50 (8)°. Further, weak C15—H15···π interaction involving phenyl (C24—C29) ring and C25—H25···π interaction involving (C4/C5/C6/C11/C12/C17) ring are found in the crystal structure (Table 1).

### Experimental

Pure 1,10-Phenanthroline-5,6-dione (2.10 g, 10 mmol) in ethanol (10 ml), aniline (0.91 g, 10 mmol), ammonium acetate (0.77 g, 10 mmol) and benzaldehyde (1.0 g, 10 mmol) was added about 1 h by maintaining the temperature at 333 K. The reaction mixture was refluxed for 7 days and extracted with dichloromethane. The solid separated was purified by column chromatography using Benzene: Ethyl acetate as the eluent. Yield: 1.48 g (40%).  Crystals suitable for X-ray diffraction studies were grown by slow solvent evaporation of a solution of the compound in dichloromethane.

### Refinement

H atoms were positioned geometrically and allowed to ride on their parent atoms, with C—H = 0.95 Å; *U*_iso_(H) = 1.2*U*_eq_(C).

### Crystal data

**Table:**

C_25_H_16_N_4_	*Z* = 2
*M**_r_* = 372.42	*F*(000) = 388
Triclinic, *P*1	*D*_x_ = 1.323 Mg m^−^^3^
Hall symbol: -P 1	Melting point: 579 K
*a* = 8.8693 (7) Å	Cu *K*α radiation, λ = 1.54184 Å
*b* = 10.0637 (6) Å	Cell parameters from 3954 reflections
*c* = 11.8960 (9) Å	θ = 4.7–70.6°
α = 100.219 (6)°	µ = 0.63 mm^−^^1^
β = 110.310 (7)°	*T* = 170 K
γ = 102.475 (6)°	Block, colourless
*V* = 934.63 (14) Å^3^	0.43 × 0.38 × 0.26 mm

### Data collection

**Table:**

Oxford Diffraction Xcalibur Eos Gemini diffractometer	3522 independent reflections
Radiation source: Enhance (Cu) X-ray Source	3121 reflections with *I* > 2σ(*I*)
graphite	*R*_int_ = 0.014
Detector resolution: 16.1500 pixels mm^-1^	θ_max_ = 70.7°, θ_min_ = 4.7°
ω scans	*h* = −10→10
Absorption correction: multi-scan (*CrysAlis RED*; Oxford Diffraction, 2010)	*k* = −12→7
*T*_min_ = 0.965, *T*_max_ = 1.000	*l* = −13→14
5832 measured reflections	

### Refinement

**Table:**

Refinement on *F*^2^	Primary atom site location: structure-invariant direct methods
Least-squares matrix: full	Secondary atom site location: difference Fourier map
*R*[*F*^2^ > 2σ(*F*^2^)] = 0.042	Hydrogen site location: inferred from neighbouring sites
*wR*(*F*^2^) = 0.117	H-atom parameters constrained
*S* = 1.05	*w* = 1/[σ^2^(*F*_o_^2^) + (0.0649*P*)^2^ + 0.1268*P*] where *P* = (*F*_o_^2^ + 2*F*_c_^2^)/3
3522 reflections	(Δ/σ)_max_ = 0.001
262 parameters	Δρ_max_ = 0.18 e Å^−^^3^
0 restraints	Δρ_min_ = −0.17 e Å^−^^3^

### Special details

**Table:**

Geometry. Bond distances, angles *etc*. have been calculated using the rounded fractional coordinates. All su's are estimated from the variances of the (full) variance-covariance matrix. The cell e.s.d.'s are taken into account in the estimation of distances, angles and torsion angles
Refinement. Refinement of *F*^2^ against ALL reflections. The weighted *R*-factor *wR* and goodness of fit *S* are based on *F*^2^, conventional *R*-factors *R* are based on *F*, with *F* set to zero for negative *F*^2^. The threshold expression of *F*^2^ > 2σ(*F*^2^) is used only for calculating *R*-factors(gt) *etc*. and is not relevant to the choice of reflections for refinement. *R*-factors based on *F*^2^ are statistically about twice as large as those based on *F*, and *R*- factors based on ALL data will be even larger.

### Fractional atomic coordinates and isotropic or equivalent isotropic displacement parameters (Å^2^)

**Table:**

	*x*	*y*	*z*	*U*_iso_*/*U*_eq_	
N1	0.44262 (13)	0.14038 (11)	0.37008 (9)	0.0404 (3)	
N3	0.21256 (13)	0.07897 (11)	0.41050 (10)	0.0436 (3)	
N10	0.70826 (15)	0.57164 (12)	0.72960 (11)	0.0510 (4)	
N13	0.4425 (2)	0.47849 (14)	0.78916 (13)	0.0678 (5)	
C2	0.28544 (15)	0.04678 (13)	0.33444 (12)	0.0414 (4)	
C4	0.32764 (16)	0.19978 (13)	0.49979 (11)	0.0407 (4)	
C5	0.47004 (15)	0.24080 (13)	0.47676 (11)	0.0394 (3)	
C6	0.60568 (15)	0.36743 (13)	0.55079 (11)	0.0399 (3)	
C7	0.74828 (17)	0.41987 (15)	0.52720 (13)	0.0503 (4)	
C8	0.86537 (19)	0.54488 (16)	0.60380 (14)	0.0555 (5)	
C9	0.83894 (19)	0.61654 (15)	0.70255 (13)	0.0546 (4)	
C11	0.59107 (17)	0.44797 (13)	0.65524 (11)	0.0425 (4)	
C12	0.44390 (18)	0.40051 (14)	0.68438 (12)	0.0462 (4)	
C14	0.3052 (3)	0.4386 (2)	0.81063 (19)	0.0818 (7)	
C15	0.1641 (3)	0.32423 (19)	0.73479 (18)	0.0710 (7)	
C16	0.1669 (2)	0.24145 (16)	0.63142 (15)	0.0550 (5)	
C17	0.31040 (17)	0.27871 (14)	0.60500 (12)	0.0436 (4)	
C18	0.54455 (15)	0.13773 (12)	0.29871 (11)	0.0392 (3)	
C19	0.68211 (17)	0.08736 (15)	0.33628 (14)	0.0511 (4)	
C20	0.7747 (2)	0.08003 (17)	0.26423 (16)	0.0598 (5)	
C21	0.72777 (19)	0.12065 (16)	0.15536 (15)	0.0584 (5)	
C22	0.5928 (2)	0.17260 (17)	0.12019 (14)	0.0582 (5)	
C23	0.50057 (17)	0.18306 (15)	0.19273 (13)	0.0487 (4)	
C24	0.21038 (15)	−0.07711 (13)	0.22359 (11)	0.0418 (4)	
C25	0.29258 (18)	−0.17796 (15)	0.21043 (13)	0.0515 (4)	
C26	0.2173 (2)	−0.29636 (15)	0.10929 (14)	0.0581 (5)	
C27	0.0601 (2)	−0.31457 (15)	0.01978 (13)	0.0578 (5)	
C28	−0.02154 (19)	−0.21497 (18)	0.03113 (14)	0.0599 (5)	
C29	0.05283 (17)	−0.09633 (16)	0.13260 (13)	0.0526 (4)	
H7	0.76323	0.36890	0.45861	0.0604*	
H8	0.96307	0.58201	0.58965	0.0666*	
H9	0.92067	0.70407	0.75433	0.0655*	
H14	0.30294	0.49252	0.88384	0.0983*	
H15	0.06790	0.30395	0.75426	0.0852*	
H16	0.07396	0.16076	0.57882	0.0660*	
H19	0.71247	0.05821	0.41066	0.0613*	
H20	0.87050	0.04707	0.28955	0.0718*	
H21	0.78933	0.11258	0.10446	0.0701*	
H22	0.56247	0.20157	0.04571	0.0698*	
H23	0.40854	0.22091	0.16961	0.0584*	
H25	0.40137	−0.16534	0.27149	0.0618*	
H26	0.27385	−0.36536	0.10130	0.0697*	
H27	0.00829	−0.39617	−0.04980	0.0693*	
H28	−0.12955	−0.22749	−0.03092	0.0718*	
H29	−0.00436	−0.02771	0.13996	0.0631*	

### Atomic displacement parameters (Å^2^)

**Table:**

	*U*^11^	*U*^22^	*U*^33^	*U*^12^	*U*^13^	*U*^23^
N1	0.0356 (5)	0.0448 (5)	0.0376 (5)	0.0087 (4)	0.0158 (4)	0.0053 (4)
N3	0.0372 (5)	0.0500 (6)	0.0417 (6)	0.0100 (4)	0.0173 (4)	0.0092 (5)
N10	0.0563 (7)	0.0464 (6)	0.0427 (6)	0.0103 (5)	0.0167 (5)	0.0069 (5)
N13	0.0882 (10)	0.0568 (7)	0.0632 (8)	0.0142 (7)	0.0476 (8)	0.0020 (6)
C2	0.0353 (6)	0.0468 (7)	0.0400 (6)	0.0102 (5)	0.0147 (5)	0.0106 (5)
C4	0.0393 (6)	0.0468 (7)	0.0387 (6)	0.0153 (5)	0.0172 (5)	0.0122 (5)
C5	0.0381 (6)	0.0456 (6)	0.0351 (6)	0.0145 (5)	0.0148 (5)	0.0098 (5)
C6	0.0379 (6)	0.0440 (6)	0.0367 (6)	0.0135 (5)	0.0129 (5)	0.0114 (5)
C7	0.0443 (7)	0.0549 (8)	0.0470 (7)	0.0086 (6)	0.0205 (6)	0.0060 (6)
C8	0.0461 (8)	0.0567 (8)	0.0549 (8)	0.0041 (6)	0.0197 (6)	0.0096 (7)
C9	0.0547 (8)	0.0477 (7)	0.0462 (7)	0.0039 (6)	0.0126 (6)	0.0066 (6)
C11	0.0478 (7)	0.0423 (6)	0.0361 (6)	0.0158 (5)	0.0139 (5)	0.0112 (5)
C12	0.0563 (8)	0.0456 (7)	0.0427 (7)	0.0198 (6)	0.0241 (6)	0.0122 (5)
C14	0.1087 (15)	0.0699 (11)	0.0839 (12)	0.0187 (10)	0.0718 (12)	0.0037 (9)
C15	0.0838 (12)	0.0683 (10)	0.0838 (12)	0.0221 (9)	0.0620 (11)	0.0180 (9)
C16	0.0581 (9)	0.0587 (8)	0.0605 (9)	0.0203 (7)	0.0360 (7)	0.0176 (7)
C17	0.0476 (7)	0.0483 (7)	0.0433 (7)	0.0203 (6)	0.0228 (6)	0.0160 (6)
C18	0.0362 (6)	0.0397 (6)	0.0395 (6)	0.0077 (5)	0.0179 (5)	0.0050 (5)
C19	0.0470 (7)	0.0592 (8)	0.0555 (8)	0.0211 (6)	0.0243 (6)	0.0219 (7)
C20	0.0510 (8)	0.0654 (9)	0.0765 (10)	0.0258 (7)	0.0356 (8)	0.0208 (8)
C21	0.0578 (9)	0.0624 (9)	0.0638 (9)	0.0141 (7)	0.0401 (8)	0.0112 (7)
C22	0.0611 (9)	0.0690 (9)	0.0492 (8)	0.0152 (7)	0.0288 (7)	0.0192 (7)
C23	0.0441 (7)	0.0563 (8)	0.0472 (7)	0.0166 (6)	0.0187 (6)	0.0155 (6)
C24	0.0388 (6)	0.0453 (7)	0.0379 (6)	0.0059 (5)	0.0165 (5)	0.0097 (5)
C25	0.0506 (8)	0.0487 (7)	0.0449 (7)	0.0138 (6)	0.0091 (6)	0.0105 (6)
C26	0.0726 (10)	0.0453 (7)	0.0491 (8)	0.0184 (7)	0.0167 (7)	0.0105 (6)
C27	0.0705 (10)	0.0473 (7)	0.0386 (7)	0.0035 (7)	0.0131 (7)	0.0068 (6)
C28	0.0460 (8)	0.0730 (10)	0.0430 (7)	0.0075 (7)	0.0072 (6)	0.0086 (7)
C29	0.0414 (7)	0.0639 (9)	0.0481 (7)	0.0149 (6)	0.0163 (6)	0.0094 (6)

### Geometric parameters (Å, °)

**Table:**

N1—C2	1.3752 (18)	C20—C21	1.380 (2)
N1—C5	1.3840 (16)	C21—C22	1.372 (3)
N1—C18	1.4402 (18)	C22—C23	1.386 (2)
N3—C2	1.3168 (18)	C24—C25	1.389 (2)
N3—C4	1.3789 (17)	C24—C29	1.386 (2)
N10—C9	1.317 (2)	C25—C26	1.380 (2)
N10—C11	1.3506 (18)	C26—C27	1.379 (2)
N13—C12	1.356 (2)	C27—C28	1.371 (3)
N13—C14	1.323 (3)	C28—C29	1.383 (2)
C2—C24	1.4782 (18)	C7—H7	0.9500
C4—C5	1.377 (2)	C8—H8	0.9500
C4—C17	1.4330 (19)	C9—H9	0.9500
C5—C6	1.4321 (18)	C14—H14	0.9500
C6—C7	1.403 (2)	C15—H15	0.9500
C6—C11	1.4204 (18)	C16—H16	0.9500
C7—C8	1.365 (2)	C19—H19	0.9500
C8—C9	1.385 (2)	C20—H20	0.9500
C11—C12	1.468 (2)	C21—H21	0.9500
C12—C17	1.408 (2)	C22—H22	0.9500
C14—C15	1.389 (3)	C23—H23	0.9500
C15—C16	1.369 (3)	C25—H25	0.9500
C16—C17	1.405 (2)	C26—H26	0.9500
C18—C19	1.384 (2)	C27—H27	0.9500
C18—C23	1.3745 (19)	C28—H28	0.9500
C19—C20	1.381 (2)	C29—H29	0.9500
			
N10···N13	2.716 (2)	C18···H25	2.9500
N13···C23^i^	3.290 (2)	C18···H7	2.5900
N13···N10	2.716 (2)	C19···H7	2.7600
N3···H19^ii^	2.7100	C19···H25	2.9200
N3···H16	2.7900	C20···H29^viii^	3.0900
N3···H20^iii^	2.7900	C21···H9^ix^	2.9300
N10···H23^i^	2.7900	C24···H15^v^	3.1000
N10···H27^iv^	2.9200	C27···H21^x^	3.0600
N10···H28^iv^	2.8500	C27···H9^vi^	3.0200
N13···H23^i^	2.9000	C27···H27^vii^	2.9500
C4···C8^i^	3.551 (2)	C28···H9^vi^	3.0800
C4···C9^i^	3.418 (2)	C28···H15^v^	2.9800
C5···C9^i^	3.585 (2)	C29···H15^v^	2.8900
C6···C11^i^	3.5427 (19)	H7···C18	2.5900
C7···C18	3.2173 (19)	H7···C19	2.7600
C7···C12^i^	3.532 (2)	H9···C27^iv^	3.0200
C7···C19	3.497 (2)	H9···C28^iv^	3.0800
C8···C17^i^	3.439 (2)	H9···C21^ix^	2.9300
C8···C4^i^	3.551 (2)	H9···H21^ix^	2.5900
C9···C4^i^	3.418 (2)	H15···C24^v^	3.1000
C9···C27^iv^	3.452 (2)	H15···C28^v^	2.9800
C9···C5^i^	3.585 (2)	H15···C29^v^	2.8900
C11···C26^ii^	3.521 (2)	H16···N3	2.7900
C11···C6^i^	3.5427 (19)	H19···N3^ii^	2.7100
C11···C25^ii^	3.551 (2)	H19···C4^ii^	2.9700
C12···C7^i^	3.532 (2)	H20···N3^viii^	2.7900
C15···C29^v^	3.597 (3)	H20···H29^viii^	2.4900
C17···C8^i^	3.439 (2)	H21···H29^viii^	2.5100
C18···C7	3.2173 (19)	H21···C27^x^	3.0600
C18···C25	3.211 (2)	H21···H9^ix^	2.5900
C19···C7	3.497 (2)	H23···N10^i^	2.7900
C19···C25	3.509 (2)	H23···N13^i^	2.9000
C23···C24	3.421 (2)	H23···H28^xi^	2.4700
C23···N13^i^	3.290 (2)	H25···C18	2.9500
C24···C23	3.421 (2)	H25···C19	2.9200
C25···C18	3.211 (2)	H25···C4^ii^	3.0700
C25···C19	3.509 (2)	H25···C12^ii^	3.0200
C25···C11^ii^	3.551 (2)	H25···C17^ii^	3.0400
C26···C11^ii^	3.521 (2)	H26···C11^ii^	3.0600
C27···C9^vi^	3.452 (2)	H26···C12^ii^	3.0300
C27···C27^vii^	3.549 (2)	H27···N10^vi^	2.9200
C29···C15^v^	3.597 (3)	H27···C9^vi^	2.8500
C4···H25^ii^	3.0700	H27···C27^vii^	2.9500
C4···H19^ii^	2.9700	H27···H27^vii^	2.5800
C9···H27^iv^	2.8500	H28···N10^vi^	2.8500
C11···H26^ii^	3.0600	H28···H23^xi^	2.4700
C12···H26^ii^	3.0300	H29···C20^iii^	3.0900
C12···H25^ii^	3.0200	H29···H20^iii^	2.4900
C17···H25^ii^	3.0400	H29···H21^iii^	2.5100
			
C2—N1—C5	106.40 (11)	C2—C24—C25	121.16 (12)
C2—N1—C18	124.47 (10)	C2—C24—C29	119.87 (13)
C5—N1—C18	128.80 (11)	C25—C24—C29	118.94 (13)
C2—N3—C4	104.52 (12)	C24—C25—C26	120.44 (14)
C9—N10—C11	117.80 (13)	C25—C26—C27	120.03 (16)
C12—N13—C14	116.67 (16)	C26—C27—C28	120.05 (14)
N1—C2—N3	112.63 (11)	C27—C28—C29	120.24 (15)
N1—C2—C24	122.78 (12)	C24—C29—C28	120.30 (15)
N3—C2—C24	124.59 (13)	C6—C7—H7	120.00
N3—C4—C5	111.31 (12)	C8—C7—H7	120.00
N3—C4—C17	127.30 (13)	C7—C8—H8	121.00
C5—C4—C17	121.38 (12)	C9—C8—H8	121.00
N1—C5—C4	105.14 (11)	N10—C9—H9	118.00
N1—C5—C6	131.74 (13)	C8—C9—H9	118.00
C4—C5—C6	123.04 (12)	N13—C14—H14	117.00
C5—C6—C7	125.74 (12)	C15—C14—H14	117.00
C5—C6—C11	116.56 (13)	C14—C15—H15	121.00
C7—C6—C11	117.68 (12)	C16—C15—H15	121.00
C6—C7—C8	119.33 (14)	C15—C16—H16	121.00
C7—C8—C9	118.70 (16)	C17—C16—H16	121.00
N10—C9—C8	124.49 (14)	C18—C19—H19	120.00
N10—C11—C6	121.98 (14)	C20—C19—H19	120.00
N10—C11—C12	117.39 (12)	C19—C20—H20	120.00
C6—C11—C12	120.61 (12)	C21—C20—H20	120.00
N13—C12—C11	117.44 (13)	C20—C21—H21	120.00
N13—C12—C17	122.17 (15)	C22—C21—H21	120.00
C11—C12—C17	120.40 (12)	C21—C22—H22	120.00
N13—C14—C15	125.3 (2)	C23—C22—H22	120.00
C14—C15—C16	118.5 (2)	C18—C23—H23	121.00
C15—C16—C17	118.39 (17)	C22—C23—H23	121.00
C4—C17—C12	117.84 (14)	C24—C25—H25	120.00
C4—C17—C16	123.29 (13)	C26—C25—H25	120.00
C12—C17—C16	118.86 (13)	C25—C26—H26	120.00
N1—C18—C19	119.73 (11)	C27—C26—H26	120.00
N1—C18—C23	118.93 (13)	C26—C27—H27	120.00
C19—C18—C23	121.33 (14)	C28—C27—H27	120.00
C18—C19—C20	119.13 (14)	C27—C28—H28	120.00
C19—C20—C21	119.88 (17)	C29—C28—H28	120.00
C20—C21—C22	120.41 (16)	C24—C29—H29	120.00
C21—C22—C23	120.35 (15)	C28—C29—H29	120.00
C18—C23—C22	118.84 (15)		
			
C5—N1—C2—N3	−0.73 (15)	C4—C5—C6—C11	2.93 (19)
C5—N1—C2—C24	−179.76 (12)	C5—C6—C7—C8	177.87 (14)
C18—N1—C2—N3	−174.58 (12)	C11—C6—C7—C8	−0.4 (2)
C18—N1—C2—C24	6.4 (2)	C5—C6—C11—N10	−177.82 (12)
C2—N1—C5—C4	0.88 (14)	C5—C6—C11—C12	0.63 (19)
C2—N1—C5—C6	−175.82 (14)	C7—C6—C11—N10	0.6 (2)
C18—N1—C5—C4	174.37 (12)	C7—C6—C11—C12	179.08 (13)
C18—N1—C5—C6	−2.3 (2)	C6—C7—C8—C9	−0.3 (2)
C2—N1—C18—C19	−104.93 (15)	C7—C8—C9—N10	0.9 (2)
C2—N1—C18—C23	73.81 (17)	N10—C11—C12—N13	−4.9 (2)
C5—N1—C18—C19	82.65 (17)	N10—C11—C12—C17	174.82 (13)
C5—N1—C18—C23	−98.62 (16)	C6—C11—C12—N13	176.55 (13)
C4—N3—C2—N1	0.24 (15)	C6—C11—C12—C17	−3.7 (2)
C4—N3—C2—C24	179.25 (12)	N13—C12—C17—C4	−177.04 (14)
C2—N3—C4—C5	0.35 (15)	N13—C12—C17—C16	3.7 (2)
C2—N3—C4—C17	−179.93 (14)	C11—C12—C17—C4	3.2 (2)
C11—N10—C9—C8	−0.7 (2)	C11—C12—C17—C16	−176.00 (14)
C9—N10—C11—C6	−0.1 (2)	N13—C14—C15—C16	2.6 (3)
C9—N10—C11—C12	−178.58 (13)	C14—C15—C16—C17	−1.9 (3)
C14—N13—C12—C11	176.58 (15)	C15—C16—C17—C4	179.78 (16)
C14—N13—C12—C17	−3.2 (2)	C15—C16—C17—C12	−1.0 (2)
C12—N13—C14—C15	0.0 (3)	N1—C18—C19—C20	177.39 (13)
N1—C2—C24—C25	56.56 (19)	C23—C18—C19—C20	−1.3 (2)
N1—C2—C24—C29	−125.55 (15)	N1—C18—C23—C22	−176.22 (13)
N3—C2—C24—C25	−122.35 (16)	C19—C18—C23—C22	2.5 (2)
N3—C2—C24—C29	55.54 (19)	C18—C19—C20—C21	−1.0 (2)
N3—C4—C5—N1	−0.78 (15)	C19—C20—C21—C22	2.1 (3)
N3—C4—C5—C6	176.28 (12)	C20—C21—C22—C23	−0.9 (3)
C17—C4—C5—N1	179.48 (12)	C21—C22—C23—C18	−1.4 (2)
C17—C4—C5—C6	−3.5 (2)	C2—C24—C25—C26	176.95 (14)
N3—C4—C17—C12	−179.46 (13)	C29—C24—C25—C26	−1.0 (2)
N3—C4—C17—C16	−0.3 (2)	C2—C24—C29—C28	−177.29 (14)
C5—C4—C17—C12	0.2 (2)	C25—C24—C29—C28	0.7 (2)
C5—C4—C17—C16	179.43 (14)	C24—C25—C26—C27	0.6 (2)
N1—C5—C6—C7	0.8 (2)	C25—C26—C27—C28	0.1 (3)
N1—C5—C6—C11	179.13 (13)	C26—C27—C28—C29	−0.4 (3)
C4—C5—C6—C7	−175.39 (14)	C27—C28—C29—C24	0.0 (2)

Symmetry codes: (i) −*x*+1, −*y*+1, −*z*+1; (ii) −*x*+1, −*y*, −*z*+1; (iii) *x*−1, *y*, *z*; (iv) *x*+1, *y*+1, *z*+1; (v) −*x*, −*y*, −*z*+1; (vi) *x*−1, *y*−1, *z*−1; (vii) −*x*, −*y*−1, −*z*; (viii) *x*+1, *y*, *z*; (ix) −*x*+2, −*y*+1, −*z*+1; (x) −*x*+1, −*y*, −*z*; (xi) −*x*, −*y*, −*z*.

### Hydrogen-bond geometry (Å, °)

**Table:**

Cg4 and Cg6 are the centroids of the C4–C6/C11/C12/C17 and C24–C29 rings, respectively.

**Table:**

*D*—H···*A*	*D*—H	H···*A*	*D*···*A*	*D*—H···*A*
C15—H15···Cg6^v^	0.95	2.86	3.757 (3)	157
C25—H25···Cg4^ii^	0.95	2.75	3.4835 (16)	135

Symmetry codes: (v) −*x*, −*y*, −*z*+1; (ii) −*x*+1, −*y*, −*z*+1.
